# Advances in the Molecular Pathogenesis and Targeted Therapy of Psoriasis: Molecular Pathogenesis of Psoriasis

**DOI:** 10.31661/gmj.vi.3854

**Published:** 2025-05-11

**Authors:** Parivash Shokoufa, Mahsa Aghaei

**Affiliations:** ^1^ Azad University of Tonekabon, Faculty of Medicine, Tonekabon, Iran; ^2^ Islamic Azad University of Medical Sciences, Tehran, Iran

**Keywords:** Psoriasis, Immune Dysregulation, Genetic Alterations, Epigenetics, Targeted Therapy, IL-17/IL-23 Inhibitors, Precision Medicin

## Abstract

Psoriasis, a chronic immune-mediated skin disorder affecting 2-3% of the global population, is driven by a complex interplay of immune dysregulation, keratinocyte dysfunction, and genetic/epigenetic alterations, with systemic comorbidities like psoriatic arthritis and cardiovascular disease amplifying its burden. Recent molecular insights, leveraging single-cell RNA sequencing and transcriptomics, have elucidated key pathogenic mechanisms, including GSDME-mediated pyroptosis, IL-23/IL-17 axis activation, YAP1-driven proliferation, and epigenetic modulation via miR-106a-5p and lncRNA MEG3. These findings have spurred targeted therapies: IL-17 inhibitors (e.g., secukinumab) achieve rapid histologic remission, IL-23 inhibitors (e.g., risankizumab) offer sustained efficacy, and novel approaches like hyperforin, Deu@Cal microneedles, and concentrated growth factor (CGF) target diverse pathways in preclinical and early clinical settings. However, challenges persist, including adverse events (e.g., paradoxical eczema, MACEs), treatment resistance (81% biologic switching), and gaps in personalization despite promising biomarkers (e.g., calprotectin, miR-106a-5p). Future directions emphasize multi-omics integration, novel agents, and combination therapies to overcome these hurdles, aiming to transform psoriasis management into a paradigm of precision medicine.

## Introduction

**Figure-1:**
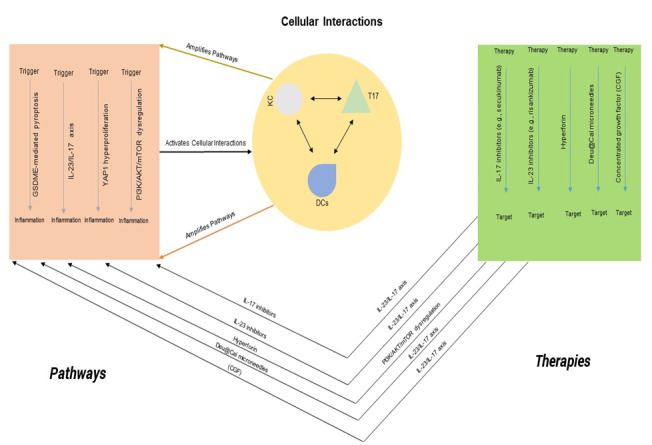
Figure 1. Integrated Model of Psoriasis Pathogenesis and Targeted Therapies. Left: Key molecular pathways driving psoriasis, including GSDME-mediated pyroptosis, IL-23/IL-17 axis, YAP1 hyperproliferation, and PI3K/AKT/mTOR dysregulation. Center: Cellular interactions between keratinocytes (KC), T17 cells, and dendritic cells (DCs) amplifying inflammation. Right: Targeted therapies inhibiting specific pathways to restore skin homeostasis. Arrows indicate interactions or therapeutic targets, with references noted.

Psoriasis, a chronic immune-mediated inflammatory skin disorder, affects approximately 2-3% of the global population, presenting as erythematous, scaly plaques that profoundly impact quality of life [1]. Beyond its cutaneous manifestations, psoriasis is increasingly recognized as a systemic disease, intertwined with comorbidities such as psoriatic arthritis (affecting 20-30% of patients), cardiovascular disease, metabolic syndrome, and latent tuberculosis infection (LTBI), which collectively escalate its clinical, psychological, and socioeconomic toll [2][3][4][5]. The past decade has ushered in a revolutionary understanding of psoriasis, propelled by cutting-edge technologies like single-cell RNA sequencing (scRNAseq), spatial transcriptomics, and proteomic profiling, which have decoded its molecular intricacies [6][7][8]. These advancements reveal a dynamic interplay of immune dysregulation, aberrant keratinocyte proliferation, and genetic/epigenetic alterations as the disease’s driving forces [9][10]. Key pathogenic pathways—including the IL-23/IL-17 axis, GSDME-mediated pyroptosis, YAP1-induced hyperproliferation, and epigenetic modulation via miR-106a-5p and lncRNA MEG3—have emerged as critical mediators of inflammation and epidermal pathology [11][12][13] (see Figure-1). This review synthesizes findings from peer-reviewed studies published up to March 2025, sourced from PubMed, GEO datasets (e.g., GSE41662), and clinical trial registries (e.g., ClinicalTrials.gov NCT03611751), to explore the molecular pathogenesis and therapeutic innovations transforming psoriasis management.

These molecular breakthroughs have catalyzed a shift from nonspecific immunosuppression to precision-targeted therapies, reshaping psoriasis treatment. Biologics such as secukinumab (an IL-17 inhibitor) and risankizumab (an IL-23 inhibitor) have set new benchmarks, delivering rapid histologic remission and sustained efficacy in diverse patient cohorts [14]. Meanwhile, novel approaches like hyperforin, Deu@Cal microneedles, and concentrated growth factor (CGF) are expanding the therapeutic arsenal by targeting distinct pathways in preclinical and early clinical stages [15]. Yet, challenges persist: adverse events (e.g., paradoxical eczema, major adverse cardiovascular events [MACEs]), treatment resistance (e.g., 81% biologic switching), and gaps in personalization despite promising biomarkers like calprotectin and miR-106a-5p underscore the need for continued innovation [2][3]. This review aims to consolidate recent advances in the molecular underpinnings of psoriasis, assess the efficacy and limitations of targeted therapies, and outline future directions to bridge these gaps, paving the way for a precision medicine paradigm tailored to individual patient profiles.

## Unveiling Psoriasis: From Clinical Burden to Molecular Insights

Psoriasis is a chronic, immune-mediated inflammatory skin disorder affecting 2-3% of the global population, marked by erythematous plaques, scaling, and significant quality-of-life impairment. Its systemic nature includes comorbidities like psoriatic arthritis, cardiovascular disease, and latent tuberculosis infection (LTBI), amplifying its clinical burden [16][17]. Molecular biology advances reveal a complex interplay of immune dysregulation, genetic predisposition, and environmental triggers. Single-cell RNA sequencing (scRNAseq) and spatial transcriptomics (STseq) highlight key cellular drivers—keratinocytes, T cells, and fibroblasts [18][19]. SLC35E1, enriched in psoriatic suprabasal layers, mitigates IMQ-induced hyperplasia when deficient [20]. YAP1 upregulation drives keratinocyte proliferation and inflammation, correlating with severity [11]. Gasdermin E (GSDME)-mediated pyroptosis, triggered by caspase-3 and TNF-α, amplifies psoriatic inflammation, with its inhibition reducing lesions in IMQ models [21]. SerpinB7 elevation suggests a protective role, as its deficiency worsens hyperplasia [22]. Epigenetically, miR-106a-5p upregulation correlates with PASI scores and cytokines [23]. Targeted therapies capitalize on these insights: secukinumab resolves histologic and transcriptomic features [24], risankizumab modulates early cellular responses [18], and hyperforin reduces IMQ-induced lesions via MAPK/STAT3 [25]. LncRNA MEG3 suppresses PI3K/AKT/mTOR, enhancing autophagy [13], while microneedle-delivered Deu@Cal alleviates hyperplasia and inflammation in IMQ models [15]. Concentrated growth factor (CGF) reduces IL-17 and improves persistent lesions in IL-23-treated patients [26]. This review synthesizes these advances (summarized in Figure-1 and Table-1), offering a roadmap for future psoriasis research and therapy.

## Decoding the Molecular Puzzle of Psoriasis: Immunity, Genetics, and Beyond

**Table:** Table1. Comparative Overview of Molecular Pathogenesis and Targeted Therapies in Psoriasis

**Aspect**	**Molecular Mechanism/Pathway**	**Key Findings**	**Therapeutic Effects**	**Biomarkers**	**Preclinical vs. Clinical**	**Limitations/Challenges**
Immune Dysregulation	GSDME-mediated pyroptosis [22]	TNF-α/caspase-3 activates GSDME, releasing IL-1β, IL-6, TNF-α in keratinocytes	Gsdme knockout or caspase-3 inhibition reduces inflammation in IMQ models	GSDME, IL-1β, IL-6 expression	Preclinical (mice, in vitro)	Limited human data; needs validation
	IL-23/IL-17 axis [14][18][23]	IL-17A/F upregulation in T17 cells; IL-23 from DCs amplifies inflammation	Risankizumab, Deu@Cal MNs reduce IL-17 signaling and cytokines	IL-17A/F, CXCL13 (T17 activity)	Clinical (risankizumab), Preclinical (Deu@Cal)	Paradoxical eczema (IL-17) [13]
	PI3K/AKT/mTOR signaling [17]	LncRNA MEG3 suppresses pathway, reducing IL-6, IL-8, IFN-γ	Enhances autophagy, reduces inflammation in TNF-α-treated cells and IMQ mice	p-PI3K, p-AKT, LC3 (autophagy)	Preclinical (mice, in vitro)	Preclinical stage; needs human trials
Keratinocyte Defects	SLC35E1 proliferation control [1]	Enriched in suprabasal layer; knockout reduces Ki67+ cells	Mitigates IMQ-induced hyperplasia	Ki67, EdU (proliferation)	Preclinical (mice)	Mechanism not fully elucidated
	YAP1 hyperproliferation [5]	Upregulated via STAT3/NF-κB, correlates with severity	Potential target; no specific therapy yet	YAP1, STAT3 (severity)	Preclinical (human samples)	Lack of targeted inhibitors
	Antimicrobial peptides (AMPs) [16]	S100A7-9, CRAMP upregulated in IMQ lesions	Hyperforin reduces AMPs, alleviating lesions	S100A7-9, CRAMP	Preclinical (mice, in vitro)	Limited to preclinical models
Targeted Therapies	IL-17 inhibitors (e.g., secukinumab) [12][15][21]	Blocks IL-17A, reduces T17 signatures and KC hyperplasia	PASI75 in 62.5% by week 12; histologic reversal in 56.5%	IL-17A, KRT16, Ki67	Clinical	Paradoxical eczema (1.22/100,000 person-years) [13]
	IL-23 inhibitors (e.g., risankizumab) [14][15][19]	Inhibits IL-23, modulates myeloid cells and fibroblasts	PASI ≤ 3 in 89% at week 52; lowest switch rate (8.5% at 24 months)	WNT5A+/IL24+ fibroblasts, IL-23	Clinical	MACE risk with IL-12/23 inhibitors [20]
	Hyperforin [16]	Suppresses MAPK/STAT3, reduces γδ T cells and AMPs	Comparable to methotrexate in IMQ models	TNF-α, IL-17A, S100A7-9	Preclinical (mice, in vitro)	Preclinical; scalability unclear
	Deu@Cal Microneedles [23]	Dual release: Deu (TYK2 inhibition), Cal (hyperplasia reduction)	Reduces PASI, Ki67, and cytokines (TNF-α, IL-23, IL-17) in IMQ mice	Ki67, IL-17, TNF-α	Preclinical (mice)	Early-stage; needs clinical trials
	Concentrated Growth Factor (CGF) [24]	Downregulates IL-17, enhances skin barrier	Improves persistent lesions in IL-23-treated patients; reduces epidermal thickness	Peripheral IL-17, Il20, Cxcl5	Clinical (small cohort)	Small sample size; mechanism unclear
	JAK inhibitors (e.g., tofacitinib) [25]	Targets JAK/STAT signaling in resistant cases	Effective in 3/3 patients with arthritis after biologic failures	None identified	Clinical (small cohort)	Limited data; long-term safety unknown
Immune Dysregulation	GSDME-mediated pyroptosis [22]	TNF-α/caspase-3 activates GSDME, releasing IL-1β, IL-6, TNF-α in keratinocytes	Gsdme knockout or caspase-3 inhibition reduces inflammation in IMQ models	GSDME, IL-1β, IL-6 expression	Preclinical (mice, in vitro)	Limited human data; needs validation
	IL-23/IL-17 axis [14][18][23]	IL-17A/F upregulation in T17 cells; IL-23 from DCs amplifies inflammation	Risankizumab, Deu@Cal MNs reduce IL-17 signaling and cytokines	IL-17A/F, CXCL13 (T17 activity)	Clinical (risankizumab), Preclinical (Deu@Cal)	Paradoxical eczema (IL-17) [13]
	PI3K/AKT/mTOR signaling [17]	LncRNA MEG3 suppresses pathway, reducing IL-6, IL-8, IFN-γ	Enhances autophagy, reduces inflammation in TNF-α-treated cells and IMQ mice	p-PI3K, p-AKT, LC3 (autophagy)	Preclinical (mice, in vitro)	Preclinical stage; needs human trials
Keratinocyte Defects	SLC35E1 proliferation control [1]	Enriched in suprabasal layer; knockout reduces Ki67+ cells	Mitigates IMQ-induced hyperplasia	Ki67, EdU (proliferation)	Preclinical (mice)	Mechanism not fully elucidated
	YAP1 hyperproliferation [5]	Upregulated via STAT3/NF-κB, correlates with severity	Potential target; no specific therapy yet	YAP1, STAT3 (severity)	Preclinical (human samples)	Lack of targeted inhibitors
	Antimicrobial peptides (AMPs) [16]	S100A7-9, CRAMP upregulated in IMQ lesions	Hyperforin reduces AMPs, alleviating lesions	S100A7-9, CRAMP	Preclinical (mice, in vitro)	Limited to preclinical models

Psoriasis pathogenesis integrates immune dysregulation, keratinocyte defects, and genetic/epigenetic factors.

### Immune Dysregulation and Inflammatory Pathways

Innate and adaptive immunity drive psoriasis. GSDME-mediated pyroptosis in keratinocytes, activated by caspase-3 and TNF-α, releases IL-1β, IL-6, and TNF-α in psoriatic lesions and IMQ models, with Gsdme knockout or caspase-3 inhibition reducing inflammation [21]. SerpinB7 deficiency exacerbates IMQ-induced cytokines (TNF-α, IL-1β, IL-23) and neutrophil infiltration [22]. WGCNA links OXSM to gamma-delta T-cell infiltration [27]. Risankizumab reduces IL-17 signaling in keratinocytes and modulates myeloid cells [18]. Hyperforin suppresses splenic γδ T cells and cytokines (TNF-α, IL-6, IL-17A) via MAPK/STAT3 [25]. LncRNA MEG3 inhibits PI3K/AKT/mTOR, reducing IL-6, IL-8, IFN-γ, and IL-1β while enhancing autophagy [13]. IL-17A blockade decreases T17 signatures (IL17A, IL17F) and boosts regulatory signals (IL34, IL37) [28]. Deu@Cal microneedles downregulate TNF-α, IL-23, IL-17, and IL-6, alleviating splenomegaly in IMQ models [15]. CGF reduces peripheral and cutaneous IL-17, downregulating Il20 and Cxcl5 [26]. These findings emphasize cytokine-driven inflammation (see Figure-1).

### Keratinocyte Proliferation and Differentiation Defects

Psoriatic keratinocytes exhibit hyperproliferation and differentiation deficits. SLC35E1 knockout reduces proliferation markers (EdU, Ki67) in IMQ models [20]. SerpinB7 deficiency suppresses differentiation markers (KRT10, filaggrin) [22]. LRRC8A disruption impairs early differentiation [29]. Proteomic analysis shows reduced KRT17 and elevated elafin [30]. Secukinumab normalizes keratin-16 [24]. Hyperforin reduces antimicrobial peptides (S100A7-9, CRAMP) [25]. IL-17A blockade decreases IL-17-driven mediators (IL36G, S100A8) and increases KRT15 in basal keratinocytes [28]. Deu@Cal microneedles reduce Ki67 and epidermal thickness (ETmin: 55.8 µm vs. 123.8 µm in untreated) [15]. CGF enhances skin barrier function in IMQ models [26]. These studies clarify epidermal pathology.

### Genetic and Epigenetic Contributions

Genetic and epigenetic alterations play a pivotal role in amplifying psoriasis pathogenesis, influencing both immune activation and keratinocyte dysfunction. Transcriptomic analysis reveals 1,608 differentially expressed genes (DEGs) in psoriatic skin, enriched in actin cytoskeleton organization and cytokine-cytokine receptor interaction pathways, with genes like OXSM and ACTN4 implicated in immune cell infiltration and structural changes [31]. Weighted gene co-expression network analysis (WGCNA) further identifies OXSM as a hub gene associated with gamma-delta T-cell activity, suggesting a genetic basis for immune dysregulation [31]. YAP1, a key regulator in the Hippo signaling pathway, is significantly upregulated in psoriatic keratinocytes, driving proliferation and inflammation through STAT3 and NF-κB activation, with its expression levels strongly correlating with disease severity (e.g., PASI scores) [11]. However, YAP1’s role remains debated, as some studies suggest its inhibition alone does not fully reverse hyperplasia, indicating potential compensatory pathways [11]. Epigenetic modifications add another layer of complexity: miR-106a-5p, a microRNA overexpressed in psoriasis patients’ serum, targets PTEN, a negative regulator of the PI3K/AKT pathway, leading to enhanced signaling (AUC 0.901 for diagnostic accuracy) and increased production of inflammatory cytokines like IL-6 and TNF-α, which correlate with clinical severity [23]. Secukinumab treatment corrects approximately 68% of the dysregulated psoriasis transcriptome, reversing aberrant gene expression profiles and normalizing pathways like IL-17 signaling, demonstrating the therapeutic potential of targeting these genetic alterations [24]. Long non-coding RNA (lncRNA) MEG3 suppresses the PI3K/AKT/mTOR pathway in TNF-α-treated keratinocytes and IMQ-induced mouse models, reducing inflammatory cytokine expression (IL-6, IL-8, IFN-γ) while promoting autophagy through increased LC3 levels, highlighting its role as an epigenetic modulator [13]. Deu@Cal microneedles, delivering dual TYK2 inhibition and calcitriol, downregulate critical inflammatory pathways, including IL-17, IL-22, and type I interferon (IFN) signaling, with RNA sequencing showing 5,120 DEGs (2,692 upregulated, 2,428 downregulated) in treated IMQ mice, indicating broad transcriptomic modulation [15]. Concentrated growth factor (CGF) treatment upregulates genes associated with skin barrier function and hair cycle regulation (e.g., via GO enrichment analysis), while suppressing inflammation-associated genes like Il20 and Cxcl5 in IMQ models, suggesting an epigenetic influence on tissue repair [26]. Additionally, gasdermin E (GSDME) expression is significantly elevated in psoriatic lesions compared to non-lesional skin (GEO dataset GSE41662), but not GSDMD, pointing to a specific genetic contribution to pyroptosis-mediated inflammation; yet, conflicting data on GSDME’s dominance over other gasdermins warrant further investigation [21]. These genetic and epigenetic changes collectively fuel the inflammatory and proliferative cascades of psoriasis, offering multiple targets for precision therapeutics, though their interplay with environmental triggers and comorbidities (e.g., obesity in 48% of patients [32]) remains underexplored.

## Targeted Therapies: Precision Medicine in Psoriasis

Precision medicine targets specific pathways for psoriasis remission (summarized in Table-1).

### IL-17 and IL-23 Inhibitors: Biologic Precision

Secukinumab achieves histologic reversal in 56.5% and PASI75 in 62.5% by week 12, normalizing 68% of the transcriptome [24]. Risankizumab reduces IL-17 signaling early [18]. IL-17 inhibitors outperform IL-23 inhibitors in PASI90 at week 16 (56% vs. 42%), but IL-23 inhibitors excel in drug survival (88% vs. 75% at 24 months) and PASI ≤ 3 at week 52 (89% vs. 83%) [33]. IL-23 inhibitors show the lowest switch rates (12.7% at 24 months) vs. TNF inhibitors (39.1%) [34]. IL-17 inhibitors carry a higher paradoxical eczema risk (1.22 vs. 0.56 per 100,000 person-years) [35], and IL-12/23 inhibitors link to MACEs (PRR 518.28 for myocardial infarction) [16]. In LTBI patients, IL-17/IL-23 inhibitors show low TB reactivation (0.46% for IL-17, 0% for IL-23), even without full prophylaxis [17]. Ustekinumab and tofacitinib succeed in treatment-resistant cases [32]. These data highlight efficacy-safety balances; however, IL-17 inhibitors’ failure in some patients (e.g., 37.5% not achieving PASI75 by week 12 [24]) may stem from compensatory IL-23 or TNF-α activity, necessitating combination strategies or alternative targets [33] .

### Small Molecules and Conventional Therapies

Methotrexate reduces PASI (21.93 to 11.20) and calprotectin (83.22 to 59.04 ng/ml) [36]. MMFAL curbs hyperplasia in IMQ models [37]. Hyperforin matches methotrexate in reducing IMQ lesions via MAPK/STAT3 [25]. LncRNA MEG3 enhances autophagy via PI3K/AKT/mTOR inhibition [13]. Deu@Cal microneedles outperform single-drug MNs, reducing PASI and cytokines [15]. CGF improves persistent lesions in IL-23-treated patients [26]. These complement biologics.

### Personalized Approaches and Biomarkers

Calprotectin predicts methotrexate response (cutoff 60 ng/ml, sensitivity 82.35%) [36]. MiR-106a-5p (AUC 0.901) tracks inflammation [23]. WNT5A+/IL24+ fibroblasts guide IL-23 therapy [18]. Peripheral IL-17 reduction post-CGF correlates with lesion improvement [26]. Multi-omics could refine personalization.

## Challenges and Future Directions

Despite significant advances in understanding psoriasis pathogenesis and developing targeted therapies, several challenges remain that necessitate innovative solutions and forward-looking strategies to improve patient outcomes (see Table-1 for a comparative overview).

### Therapeutic Limitations and Adverse Events

Current therapies, while effective, face limitations that impact their long-term utility. IL-17 inhibitors like secukinumab deliver rapid PASI90 responses (56% at week 16), yet their association with paradoxical eczema (1.22 per 100,000 person-years) complicates their use in susceptible patients [13]. IL-12/23 inhibitors, such as ustekinumab, exhibit the highest risk of major adverse cardiovascular events (MACEs), with a PRR of 518.28 for myocardial infarction, raising safety concerns, particularly in patients with cardiovascular comorbidities [20]. TNF inhibitors, despite historical efficacy, show the highest switch rates (39.1% at 24 months), indicating reduced durability and potential loss of response over time, often due to immunogenicity or mechanistic failure [19]. In patients with LTBI, biologic therapies like IL-17 and IL-23 inhibitors demonstrate low TB reactivation rates (0.46% and 0%, respectively), but incomplete chemoprophylaxis—interrupted in 75.6% of cases due to hepatotoxicity—poses a persistent risk, especially in high-burden regions [21]. Treatment resistance is another hurdle, with 81% of patients switching from their index biologic, predominantly due to primary failure (21/29 cases), suggesting underlying mechanistic mismatches rather than secondary immunogenicity [25]. Emerging therapies like hyperforin, lncRNA MEG3, and Deu@Cal microneedles show promise in preclinical models by targeting MAPK/STAT3, PI3K/AKT/mTOR, and IL-23/IL-17 pathways, respectively, but their translation to clinical practice is hindered by a lack of human trials and scalability challenges [16][17][23]. Addressing these adverse events and resistance patterns requires a deeper understanding of patient-specific factors and the development of safer, more durable therapeutic options.

### Personalization and Predictive Tools

The heterogeneity of psoriasis responses underscores the urgent need for personalized treatment strategies, yet current tools remain insufficiently robust. Biomarkers like serum calprotectin (cutoff 60 ng/ml, sensitivity 82.35%) and miR-106a-5p (AUC 0.901) effectively predict methotrexate response and inflammatory activity, respectively, but their application is limited to specific contexts [10][11]. Single-cell RNA sequencing has identified WNT5A+/IL24+ fibroblasts as a therapeutic target for IL-23 inhibitors like risankizumab, offering a glimpse into cellular-level precision [14]. Similarly, CGF treatment’s reduction of peripheral IL-17 correlates with clinical improvement in resistant lesions, suggesting its potential as a dynamic biomarker [24]. However, these markers are fragmented, and no unified panel integrates genomic, transcriptomic, and proteomic data to predict outcomes across therapies. The complexity of translating scRNAseq findings into routine diagnostics—due to cost, technical expertise, and standardization issues—further delays progress. Artificial intelligence (AI) and machine learning hold promise for analyzing multi-omics data to stratify patients, as demonstrated in other fields like oncology, but their application in psoriasis is nascent. Developing comprehensive, accessible predictive tools that account for genetic, immune, and environmental variables remains a critical challenge to achieving true precision medicine.

### Future Therapeutic Horizons

The future of psoriasis management lies in innovative therapies and integrative approaches that address current gaps. Preclinical candidates like hyperforin, which suppresses MAPK/STAT3 and rivals methotrexate in IMQ models, and lncRNA MEG3, which enhances autophagy via PI3K/AKT/mTOR inhibition, offer novel mechanisms that could bypass resistance to existing biologics [16][17]. Deu@Cal microneedles, with dual TYK2 inhibition and hyperplasia reduction, demonstrate superior PASI reduction in IMQ mice, suggesting a platform for localized, sustained delivery that minimizes systemic side effects [23]. CGF, by downregulating IL-17 and enhancing skin barrier function, provides a complementary approach for resistant lesions, as seen in IL-23-treated patients [24]. IL-23 inhibitors like risankizumab, with an 8.5% switch rate at 24 months, set a benchmark for efficacy and safety, yet their cardiovascular profile needs refinement, especially given IL-12/23 inhibitors’ MACE risks [19][20]. JAK inhibitors like tofacitinib succeed in treatment-resistant cases with arthritis, achieving responses in 3/3 patients after multiple biologic failures, but their long-term safety profile remains understudied [25]. Integrating AI-driven predictive models with single-cell omics could redefine psoriasis endotypes, enabling precision therapies tailored to individual molecular signatures—an approach already transforming oncology and poised to revolutionize dermatology [17]. To mitigate risks like MACEs and TB reactivation [20][21], next-generation agents must prioritize specificity and reduced off-target effects. Combination therapies—integrating biologics (e.g., IL-23 inhibitors), small molecules (e.g., hyperforin), and lifestyle interventions (e.g., diet, stress management)—could synergistically target multiple pathways, improving efficacy while reducing adverse events. Clinical trials exploring these combinations, alongside RNA-based therapies (e.g., lncRNA MEG3 mimics) and advanced delivery systems (e.g., microneedles), are essential to translate preclinical promise into patient benefit.

## Conclusion

Recent advances in psoriasis research have illuminated a complex molecular landscape, pinpointing immune dysregulation (e.g., GSDME-mediated pyroptosis, IL-23/IL-17 axis), keratinocyte dysfunction (e.g., SLC35E1, YAP1), and epigenetic regulation (e.g., miR-106a-5p, lncRNA MEG3) as central drivers of its pathogenesis [25][35][36]. These discoveries have spurred the development of targeted therapies—biologics like secukinumab and risankizumab, small molecules like hyperforin, and innovative modalities such as Deu@Cal microneedles and concentrated growth factor (CGF)—that deliver unprecedented precision in disease management [40][41] (see Figure-1 and Table-1). However, significant hurdles remain. Adverse events, including cardiovascular risks with IL-12/23 inhibitors and TB reactivation in LTBI patients, alongside high treatment resistance rates (e.g., 81% biologic switching), highlight the limitations of current options [27][28][29]. Moreover, the promise of personalized medicine remains unfulfilled due to fragmented predictive tools, despite advances in biomarkers and multi-omics profiling [31][38].

Looking forward, the integration of artificial intelligence (AI) with single-cell omics offers a transformative opportunity to redefine psoriasis endotypes, enabling therapies tailored to individual molecular signatures—a strategy already revolutionizing fields like oncology [42]. Future efforts must prioritize the development of next-generation agents with enhanced safety profiles, such as selective JAK inhibitors or RNA-based therapies (e.g., lncRNA mimics), alongside scalable combination strategies that integrate biologics, small molecules, and lifestyle interventions to target multiple disease pathways synergistically [43]. Expanding clinical trials to validate preclinical candidates like Deu@Cal microneedles and CGF, coupled with AI-driven predictive models, could bridge the gap between molecular insights and patient outcomes [41][44]. By harnessing these innovations, psoriasis management stands on the cusp of becoming a gold standard for precision medicine, delivering durable remission and improved quality of life for patients globally.

## Conflict of Interest

There are no conflicts of interest associated with this manuscript.

## References

[R1] Goldminz AM, Au S, Kim N, Gottlieb AB, Lizzul PF (2013). NF-κB: an essential transcription factor in psoriasis. Journal of dermatological science.

[R2] Garshick MS, Ward NL, Krueger JG, Berger JS (2021). Cardiovascular Risk in Patients With Psoriasis: JACC Review Topic
of the
Week. J Am Coll Cardiol.

[R3] Lupea-Chilom DS, Solovan CS, Farcas SS, Gogulescu A, Andreescu NI (2023). Latent Tuberculosis in Psoriasis Patients on Biologic Therapies:
Real-World Data from a Care Center in Romania. Medicina.

[R4] Takeshita J, Grewal S, Langan SM, Mehta NN, Ogdie A, Van Voorhees, Gelfand JM (2017). Psoriasis and comorbid diseases: Epidemiology. J Am Acad Dermatol.

[R5] Thein D, Rosenø NAL, Maul JT, Wu JJ, Skov L, Bryld LE (2023). Drug Survival of Adalimumab, Secukinumab, and Ustekinumab in
Psoriasis as
Determined by Either Dose Escalation or Drug Discontinuation during the
First 3
Years of Treatment - a Nationwide Cohort Study. J Invest Dermatol.

[R6] Gniadecki R, Osman M, Hennesey D, O'Keefe S, Thomsen SF, Iyer A (2023). Architecture of skin inflammation in psoriasis revealed by
spatial
transcriptomics. Clin Immunol.

[R7] Guo D, Li X, Wang J, Liu X, Wang Y, Huang S, Dang N (2024). Single-cell RNA-seq reveals keratinocyte and fibroblast
heterogeneity and
their crosstalk via epithelial-mesenchymal transition in psoriasis. Cell Death Dis.

[R8] Sobolev VV, Soboleva AG, Denisova EV, Pechatnikova EA, Dvoryankova E, Korsunskaya IM, Mezentsev A (2022). Proteomic Studies of Psoriasis. Biomedicines.

[R9] Dopytalska K, Ciechanowicz P, Wiszniewski K, Szymańska E, Walecka I (2021). The Role of Epigenetic Factors in Psoriasis. Int J Mol Sci.

[R10] Nestle FO, Kaplan DH, Barker J (2009). Psoriasis. N Engl J Med.

[R11] Huang C, Li W, Shen C, Jiang B, Zhang K, Li X (2025). YAP1 facilitates the pathogenesis of psoriasis via modulating
keratinocyte proliferation and inflammation. Cell Death Dis.

[R12] Li Y, He Y, Yang F, Liang R, Xu W, Cheng J (2024). Gasdermin E-mediated keratinocyte pyroptosis participates in the
pathogenesis of psoriasis by promoting skin inflammation. Br J Dermatol.

[R13] Tang ZL, Zhang K, Lv SC, Xu GW, Zhang JF, Jia HY (2021). LncRNA MEG3 suppresses PI3K/AKT/mTOR signalling pathway to
enhance
autophagy and inhibit inflammation in TNF-α-treated keratinocytes and
psoriatic mice. Cytokine.

[R14] Gordon KB, Strober B, Lebwohl M, Augustin M, Blauvelt A, Poulin Y (2018). Efficacy and safety of risankizumab in moderate-to-severe plaque
psoriasis (UltIMMa-1 and UltIMMa-2): results from two double-blind,
randomised,
placebo-controlled and ustekinumab-controlled phase 3 trials. Lancet.

[R15] Wang ZY, Zhao ZQ, Sheng YJ, Chen KJ, Chen BZ, Guo XD, Cui Y (2024). Dual-Action Psoriasis Therapy: Antiproliferative and
Immunomodulatory
Effects via Self-Locking Microneedles. Adv Sci.

[R16] Ding L, Chen C, Yang Y, Zhang X (2024). Major cardiovascular events under biologic psoriasis therapies: a
19-year
real-world analysis of FAERS data. Front Immunol.

[R17] Torres T, Chiricozzi A, Puig L, Lé AM, Marzano AV, Dapavo P (2024). Treatment of Psoriasis Patients with Latent Tuberculosis Using
IL-17 and
IL-23 Inhibitors: A Retrospective, Multinational, Multicentre Study. Am J Clin Dermatol.

[R18] Francis L, McCluskey D, Ganier C, Jiang T, Du-Harpur X, Gabriel J (2024). Single-cell analysis of psoriasis resolution demonstrates an
inflammatory
fibroblast state targeted by IL-23 blockade. Nat Commun.

[R19] Zhang B, Mei J, Liao Q, Zhou S, Huang H, Liu H (2024). Multitranscriptome analysis reveals stromal cells in the
papillary dermis
to promote angiogenesis in psoriasis vulgaris. British Journal of Dermatology.

[R20] Huang T, Chen S, Ding K, Zheng B, Lv W, Wang X (2023). SLC35E1 promotes keratinocyte proliferation in psoriasis by
regulating
zinc homeostasis. Cell Death Dis.

[R21] Li Y, He Y, Yang F, Liang R, Xu W, Li Y (2024). Gasdermin E-mediated keratinocyte pyroptosis participates in the
pathogenesis of psoriasis by promoting skin inflammation. British Journal of Dermatology.

[R22] Zheng H, Gu L, Zhao F, Zhang C, Wang Z, Zhou H (2022). SerpinB7 deficiency contributes to development of psoriasis via
calcium-mediated keratinocyte differentiation dysfunction. Cell Death Dis.

[R23] Miao X, Tong X, Hu J, Wang J (2021). Diagnostic value of microRNA-106a-5p in patients with psoriasis
and its
regulatory role in inflammatory responses. Dermatologica Sinica.

[R24] Krueger JG, Wharton KA, Schlitt T, Suprun M, Torene RI, Jiang X, et al (2019). IL-17A inhibition by secukinumab induces early clinical,
histopathologic,
and molecular resolution of psoriasis. Journal of Allergy and Clinical Immunology.

[R25] Zhang S, Zhang J, Yu J, Chen X, Zhang F, Wei W (2021). Hyperforin Ameliorates Imiquimod-Induced Psoriasis-Like Murine
Skin
Inflammation by Modulating IL-17A-Producing γδ T Cells. Front Immunol.

[R26] Xiao Q, Chu W, Guo J, Gao J, Yao W, Huang M (2024). CGF therapy: bridging androgenetic alopecia observations to
psoriasis
treatment via IL-17 pathway. Stem Cell Res Ther.

[R27] Xu B, Zhang HL, Shen B, Wu JM, Shi MT, Li XD, Guo Q (2025). Identification biomarkers and therapeutic targets of
disulfidptosis-related in rheumatoid arthritis via bioinformatics, molecular
dynamics simulation, and experimental validation. Scientific Reports.

[R28] Kim J, Lee J, Li X, Kunjravia N, Rambhia D, Cueto I (2023). Multi-omics segregate different transcriptomic impacts of
anti-IL-17A
blockade on type 17 T-cells and regulatory immune cells in psoriasis skin. Front Immunol.

[R29] Jahn M, Lang V, Rauh O, Fauth T, Buerger C (2025). The Volume-Regulated Anion Channel LRRC8 is Involved in the
Initiation of
Epidermal Differentiation and is Deregulated in Psoriasis. JID Innovations.

[R30] Chagan-Yasutan H, He N, Arlud S, Wuyun S, Gao R, Bao W (2024). Unraveling the biomolecular effects of Mongolian mind-body
interactive
psychotherapy on psoriasis: An exosome proteomic analysis. JCBP.

[R31] Xu B, Zhang HL, Shen B, Wu JM, Shi MT, Li XD, Guo Q (2025). Identification biomarkers and therapeutic targets of
disulfidptosis-related in rheumatoid arthritis via bioinformatics, molecular
dynamics simulation, and experimental validation. Sci Rep.

[R32] O'Connor C, Byrne B, Roche D, O'Connell G, O'Connell M, Murphy M (2023). Biological and JAK inhibitor therapy outcomes for severe
psoriasis in
trisomy 21. J Dermatol.

[R33] Mastorino L, Dapavo P, Susca S, Cariti C, Siliquini N, Verrone A (2024). Drug survival and clinical effectiveness of secukinumab,
ixekizumab,
brodalumab, guselkumab, risankizumab, tildrakizumab for psoriasis treatment. J Dtsch Dermatol Ges.

[R34] Armstrong AW, Patel M, Li C, Garg V, Mandava MR, Wu JJ (2023). Real-world switching patterns and associated characteristics in
patients
with psoriasis treated with biologics in the United States. J Dermatolog Treat.

[R35] Al-Janabi A, Alabas OA, Yiu ZZN, Foulkes AC, Eyre S, Khan AR (2024). Risk of Paradoxical Eczema in Patients Receiving Biologics for
Psoriasis. JAMA Dermatol.

[R36] Hamza AM, Hassan EM, Donia HM, Maamon YM (2019). Serum calprotectin as a predictive biomarker in the treatment of
psoriasis vulgaris with methotrexate. Journal of the Egyptian Women’s Dermatologic Society.

[R37] Han H, Zhang G, Yang Y, Li C, Li X, Zhong L (2025). Therapeutic potential of monomethyl fumarate and aluminum ion
combination
in alleviating inflammation and oxidative stress in psoriasis. Redox Biology.

[R38] Théry C, Witwer KW, Aikawa E, Alcaraz MJ, Anderson JD, Andriantsitohaina R, et al (2018). Minimal information for studies of extracellular vesicles 2018
(MISEV2018): a position statement of the International Society for
Extracellular
Vesicles and update of the MISEV2014 guidelines. J Extracell Vesicles.

[R39] Iskandar IY, Ashcroft DM, Warren RB, Evans I, McElhone K, Owen CM, Burden AD, Smith CH, Reynolds NJ, Griffiths CE (2017). Patterns of biologic therapy use in the management of psoriasis:
cohort
study from the British Association of Dermatologists Biologic Interventions
Register
(BADBIR). British Journal of Dermatology.

[R40] Reich K, Warren RB, Lebwohl M, Gooderham M, Strober B, Langley RG, Paul C, De Cuyper, Vanvoorden V, Madden C, Cioffi C (2021). Bimekizumab versus secukinumab in plaque psoriasis. New England Journal of Medicine.

[R41] Reich K, Warren RB, Iversen L, Puig L, Pau-Charles I, Igarashi A, Ohtsuki M, Falqués M, Harmut M, Rozzo S, Lebwohl MG (2020). Long-term efficacy and safety of tildrakizumab for
moderate-to-severe
psoriasis: pooled analyses of two randomized phase III clinical trials
(reSURFACE 1
and reSURFACE 2) through 148 weeks. British Journal of Dermatology.

[R42] Huang K, Wu X, Li Y, Lv C, Yan Y, Wu Z, Zhang M, Huang W, Jiang Z, Hu K, Li M (2023). Artificial intelligence–based psoriasis severity assessment:
Real-world
study and application. Journal of medical Internet research.

[R43] Snehasis N, Zafar S, Herve N, Siri P, Ali KH (2024). Efficacy and safety of various drug combinations in treating
plaque
Psoriasis: A meta-analysis. F1000Research.

[R44] Gowda BJ, Ahmed MG, Hani U, Kesharwani P, Wahab S, Paul K (2023). Microneedles as a momentous platform for psoriasis therapy and
diagnosis:
A state-of-the-art review. International Journal of Pharmaceutics.

